# Absolute Quantification of Nucleotide Variants in Cell-Free DNA via Quantitative NGS: Clinical Application in Non-Small Cell Lung Cancer Patients

**DOI:** 10.3390/cancers17050783

**Published:** 2025-02-25

**Authors:** Guillaume Herbreteau, Marie Marcq, Chloé Sauzay, Maxime Carpentier, Elise Pierre-Noël, Elvire Pons-Tostivint, Audrey Vallée, Sandrine Théoleyre, Acya Bizieux, Jaafar Bennouna, Marc G. Denis

**Affiliations:** 1 Laboratory of Biochemistry and Molecular Genetics Platform for Cancer, Nantes University Hospital, 44093 Nantes, France; 2 Department of Pulmonology, Vendée Regional Hospital, 85000 La Roche sur Yon, France; 3 Thoracic Oncology Department, Nantes University Hospital, 44093 Nantes, France; 4 Foch Hospital, 92150 Suresnes, France

**Keywords:** quantification, ctDNA, NGS, dPCR, NSCLC

## Abstract

**Background/Objectives**: Circulating tumor DNA (ctDNA) analysis is a powerful tool for non-invasive monitoring of tumor burden and treatment response. Reliable quantification methods are critical for the effective use of ctDNA as a tumor biomarker. Digital PCR (dPCR) offers high sensitivity and quantification, but requires the prior knowledge of tumor-specific genomic alterations. Next-generation sequencing (NGS) provides a more comprehensive approach but is semi-quantitative, relying on variant allelic fraction (VAF), which can be influenced by non-tumor cell-free DNA. **Methods**: We developed a novel quantitative NGS (qNGS) method for absolute quantification of nucleotide variants, utilizing unique molecular identifiers (UMIs) and of quantification standards (QSs), short synthetic DNA sequences modified to include characteristic mutations for unique identification in sequencing data. We evaluated the performance of this method using plasma samples spiked with mutated DNA and plasma pools from cancer patients. We further applied our technique to plasma samples from four non-small cell lung cancer (NSCLC) patients enrolled in the ELUCID trial. **Results**: Our qNGS approach demonstrated robust linearity and correlation with dPCR in both spiked and patient-derived plasma samples. Notably, the analysis of clinical samples from the ELUCID trial revealed the ability of our method to simultaneously quantify multiple variants in a single plasma sample. Significant differences in ctDNA levels were observed between baseline and post-treatment samples collected after three weeks of front-line therapy. **Conclusions**: We introduce a novel qNGS method that enables the absolute quantification of ctDNA, independent of non-tumor circulating DNA variations. This technique was applied for the first time to serial samples from NSCLC patients, demonstrating its ability to simultaneously monitor multiple variants, making it a robust and versatile tool for precision oncology.

## Simple Summary

Circulating tumor DNA (ctDNA) analysis offers a non-invasive method for tracking tumor burden and treatment response. Current quantification techniques face limitations: digital PCR (dPCR) requires prior knowledge of tumor-specific alterations, while next-generation sequencing (NGS) provides broader insights but is semi-quantitative. To address these challenges, a novel quantitative NGS (qNGS) method was developed, incorporating unique molecular identifiers (UMIs) and quantification standards (QSs). This approach enables the absolute quantification of nucleotide variants. Validated using spiked plasma samples and clinical samples from the ELUCID trial, the qNGS method demonstrated strong linearity and high correlation with dPCR. It successfully quantified multiple variants from a single plasma sample and revealed significant reductions in ctDNA levels after three weeks of therapy in non-small-cell lung cancer (NSCLC) patients. This robust method, independent of tumor genotype knowledge, enhances precision oncology by enabling simultaneous monitoring of multiple ctDNA variants.

## 1. Introduction

Monitoring tumor burden is a critical aspect of cancer patient management, directly informing therapeutic decisions and enabling personalized care. Circulating tumor DNA (ctDNA) analysis has emerged as a promising, non-invasive method for assessing tumor burden [1]. Derived from malignant cells, ctDNA is a fraction of cell-free DNA in plasma that carries tumor-specific genetic or epigenetic alterations. Studies have consistently shown that ctDNA levels correlate closely with tumor burden, often detecting changes earlier than traditional radiological or clinical methods [2,3,4], and can also help understand tumor clonal structure and evolution during treatment [5,6,7].

The clinical utility of ctDNA as a biomarker relies on accurate quantification, with two principal methods currently available: digital PCR (dPCR) and next-generation sequencing (NGS). However, no consensus exists on the optimal technique for ctDNA quantification.

Digital PCR partitions samples into numerous independent reactions, each containing a small number of target DNA molecules or even a single molecule. This partitioning enables the direct and absolute quantification of ctDNA, expressed as mutated copies per milliliter of plasma. While highly sensitive and precise, dPCR is inherently a targeted method, requiring the prior knowledge of tumor-specific genomic alterations. This limitation restricts its use to a predefined set of mutations, preventing broader genomic profiling.

NGS provides a more comprehensive approach by enabling the simultaneous detection of multiple mutations without prior knowledge of tumor-specific alterations. This capability is particularly valuable for patients whose tumor genomics have not been fully characterized. However, NGS quantification relies on the variant allelic fraction (VAF), which measures the proportion of tumor-specific mutations relative to total cell-free DNA. This relative quantification is susceptible to interference from fluctuations in non-tumor cell-free DNA levels, which can occur in conditions such as inflammation, sepsis, or stress, potentially leading to inaccuracies in tumor burden assessment. Additionally, NGS involves multiple steps, including PCR amplification, DNA purification, and dilution, which may introduce biases. DNA molecules can be overrepresented due to amplification or lost during purification and dilution, preventing the direct estimation of absolute ctDNA levels [8,9,10,11,12,13].

To overcome these challenges, we developed a method known as quantitative NGS (qNGS). This approach combines the comprehensive mutation detection capabilities of NGS with the ability to quantify ctDNA in absolute terms, expressed as copies per milliliter of plasma. By integrating unique molecular identifiers (UMIs) and quantification standards (QSs), qNGS addresses the limitations of traditional sequencing methods, offering a precise and reliable solution for tumor burden monitoring in clinical practice.

UMIs are short DNA sequences, typically 8 to 16 nucleotides in length, introduced to each DNA molecule during the initial stages of NGS library preparation. Due to their random sequences and the vast number of possible combinations, each UMI uniquely labels a single DNA molecule.

Although DNA molecules undergo amplification during library preparation and may appear multiple times in the sequencing data, sequences originating from the same DNA molecule can be identified by their shared UMI sequence [14]. This technology enables an accurate count of the initial DNA molecules present in a sample before amplification.

**Figure 1:**
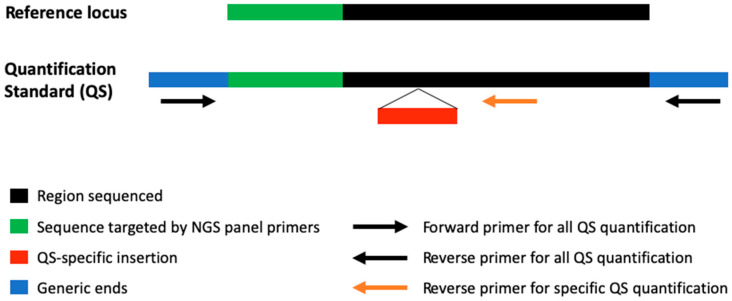
Schematic representation of quantification standards (QS). Each QS was designed based on a 103-bp reference locus. A unique 25-bp sequence was inserted adjacent to the region targeted by the NGS panel to distinguish QSs from endogenous DNA. Generics ends were introduced to allow quantification. For detailed sequences see Supplementary Figure S1.

However, not all DNA molecules initially present in a sample are represented in the sequencing data. A proportion is inevitably lost during critical steps such as cell-free DNA extraction, purification, and dilution. To address this challenge and accurately correlate the number of DNA molecules initially present in the plasma sample and the number sequenced (as determined by UMI counts), we developed QSs. QSs are synthetic DNA molecules, 190 base pairs in length, designed to mimic the size of cell-free DNA fragments, which typically result from apoptosis and constitute the majority of cell-free DNA [15]. These molecules are engineered with a sequence corresponding to a specific region of the human genome, referred to as the “reference locus”, but modified to include a characteristic mutation that distinguishes QSs from native DNA in sequencing data (Figure 1). Before cell-free DNA extraction, a known concentration of QS molecules is spiked into the plasma sample. Following extraction, the DNA is sequenced using an NGS panel capable of targeting both the reference locus and the QS sequence. Both QS molecules and the reference locus in the patient’s cell-free DNA are sequenced with comparable efficiency. The QS molecules can be identified and counted using their characteristic mutation, while UMIs enable the quantification of the original DNA molecules from the sample.

This study aimed to assess the performance of qNGS for the absolute quantification of nucleotide variants in ctDNA, offering a reliable and robust approach to addressing the inherent challenges of ctDNA analysis.

## 2. Materials and Methods

### 2.1. Quantification Standards

#### 2.1.1. QS Design

Three QSs were developed for this study. Each QS was designed based on a 103-bp reference locus selected from the GRCh38 (hg38) human reference genome. The genomic positions of the chosen reference loci are provided in the Supplementary Figure S1. To create the QS sequences, modifications were made to the reference locus sequences as follows:

Specific insertion: A unique 25-bp sequence (GATTACAACACGAGTTCGACCGCGT) was inserted adjacent to the region targeted by the NGS panel. This insertion allows the QS sequences to be easily distinguished from endogenous DNA in the sequencing data.

Generics ends: To standardize amplification, all QSs were designed with identical generic sequences at their ends, enabling their quantification with the same PCR primers during assay development. The 5′ end includes a 30-bp sequence (GTGACATCTACGGTGATCCGACATCTCCTG), and the 3′ end includes a 32-bp sequence (GTTGTTAGCATCGCCGTCATATCGCAAGGCAT).

The insertion and generic ends sequences were arbitrarily selected, and their uniqueness within the human genome was confirmed using the Basic Local Alignment Search Tool (BLAST) (https://blast.ncbi.nlm.nih.gov/Blast.cgi, accessed 24 January 2022).

The final QS sequences, each 190 bp in length, were synthesized as linear double-stranded DNA fragments using Invitrogen GenArt Strings DNA Fragments technology (ThermoFisher, Montigny-le-Bretonneux, France). The synthesized DNA fragments were then reconstituted in aqueous solution for use in this study.

#### 2.1.2. QS Quantification

The absolute quantification of each QS was performed using dPCR assays on a Naica dPCR system (Stilla Technologies, Villejuif, France). This approach utilized a universal primer set complementary to the generic ends of the QS sequences (Universal QS forward primer: GGTGATCCGACATCTCCTG; Universal QS reverse primer: ATGACGGCGATGCTAACAAC) and a Universal QS probe (HEX-GATTACAACACGAGTTCGACCGCG-TAMRA) targeting the QS-specific 25-bp insertion. This design allowed all QSs to be quantified using the same reagents.

To prepare for use, the three QSs were diluted to similar concentrations and pooled into a single solution. The pooled QSs solution was then divided into 100 µL aliquots and stored at −20 °C. To precisely determine their final concentration of each QSs within the pooled solution, triplicate dPCR assays specific to each QS were performed. These QS-specific dPCR assays utilized the same universal forward primer and labeled probe but employed unique reverse primers targeting internal sequences specific to each QSs, ensuring reaction specificity: QS1 reverse primer: AGACAGCAGATACTTGATTGGT; QS2 reverse primer: TCAATGGCTGAGGTGAGGTA; QS3 reverse primer: CCGTGCCTAGCTCAAACCTA.

#### 2.1.3. Spiking of Plasma Samples and Cell-Free DNA Extraction

For all samples analyzed in this study, 10 µL of the homogenized QS pool solution containing 18,000 copies of each QS was added to 2 mL of plasma, and immediately afterward, the lysis solution was added. Cell-free DNA was extracted using the Maxwell RSC ccfDNA LV Plasma Kit (Promega, Charbonnières-les-Bains, France) following the manufacturer’s instructions. The extracted DNA was eluted in 60 µL of elution buffer and stored at −20 °C until further use.

### 2.2. Absolute Quantification of Nucleotide Variants by qNGS

QS-spiked cell-free DNA samples were analyzed using NGS. NGS libraries were prepared with the QIAseq Targeted DNA Custom Panel kit (QIAGEN, Courtaboeuf, France), an amplicon-based library preparation method utilizing QIAseq Single Primer Extension (SPE) technology. Library preparation followed the supplier’s recommendations for cell-free DNA. The NGS panel (Supplementary Table S1) included three primers designed to target both the QSs and the corresponding reference loci in the cell-free DNA (Table 1).

NGS libraries were sequenced on a MiSeq sequencer (Illumina, Évry-Courcouronnes, France), and the resulting sequencing data were analyzed using CLC Genomics Workbench software v23 (QIAGEN) with a customized workflow. In brief, the workflow identified the QS-specific GATTACAACA CGAGTTCGACCGCGT insertion within the unaligned reads to distinguish QS sequences from those originating from the sample’s cell-free DNA. Sequences from both the QSs and the cell-free DNA were then aligned separately to the GRCh38 human reference genome (hg38):

QS sequences: These sequences mapped specifically to the corresponding reference loci. For each QS, the number of molecules sequenced was estimated based on its UMI count.

Sample cell-free DNA: After aligning the cell-free DNA sequences to the reference genome, the number of copies for each reference locus was quantified using UMI counts. Variant calling was also performed to identify any variants within the cell-free DNA.

Since QS molecules were introduced at a known concentration prior to extraction, the total cell-free DNA concentration (expressed as genomic equivalents/mL of plasma) was calculated using the following formula:

$$\left[total\;cfDNA\right]={UMI}_{ref}\times \frac{\left[QS\right]}{{UMI}_{QS}}$$

where UMI_ref_ is the UMI count of the reference locus sequences, UMI_QS_ is the UMI count of QS sequences, and [QS] represents the concentration of QS in plasma prior to extraction (copies/mL).

The quantification of each nucleotide variant (in mutated copies/mL of plasma) was calculated by applying its VAF to the total cell-free DNA concentration:

$$\left[Variant\right]=VAF\times \left[total\;cfDNA\right]$$

where VAF is the variant allele fraction of the nucleotide variant, and [total cfDNA] is the estimated total cell-free DNA concentration in plasma (genomic equivalents/mL).

Finally, the absolute quantification of each variant was determined by calculating the median of the measurements obtained from each QS.

### 2.3. Absolute Quantification of Nucleotide Variants by dPCR

EGFR exon 19 deletions, EGFR p.L858R (c.2573T > G), BRAF p.V600E (c.1799T > A) and NRAS p.Q61K (c.181C > A) variants were quantified by dPCR in QS-spiked cell-free DNA samples to assess concordance with the qNGS results.

dPCR quantification was performed using the Naica Crystal Digital PCR System (Stilla). Briefly, 1 µL of cfDNA was amplified with the PerfeCTa MultiPlex qPCR ToughMix (Quantabio, VWR International, Rosny-sous-Bois, France), 600 nM of mutation-specific PCR primers and 200 nM of TaqMan probes in a total volume of 25 µL. These samples were loaded onto Sapphire chips and amplified over 45 cycles using the Geode thermal cycler (Stilla). Fluorescence was detected using the Prism3 reader and the Crystal reader software v 4.0 (Stilla) and the absolute quantification of each variant (in mutated copies/µL of DNA sample) was performed using the Crystal Miner Software v 4.0 (Stilla).

### 2.4. Preparation of Artificial Samples

A pool of plasma from healthy donors was divided into 8 aliquots of 2 mL each. Each aliquot was spiked with 200 ng of a well-characterized, cell line-derived reference standard (OncoSpan cfDNA, Catalog ID:HD833, Horizon, Cambridge, UK). The OncoSpan standard contained 13 variants commonly found in various tumor types which are detectable by our NGS panel. These variants had known concentrations, which were validated by the supplier (Table 2). Each extract was analyzed three times, resulting in a total of 24 determined values for each of the 13 variants.

## 3. Results

### 3.1. Evaluation of qNGS on Artificial Samples

We first evaluated the qNGS method with reconstituted samples prepared by spiking normal plasma with DNA standards.

The average sequencing depth was 1016X, 1112X, and 991X for QS1, QS2, and QS3, respectively (Q1–Q3: 820–1160X; 939–1250X; 827–1112X, respectively). The sequencing depth of their reference loci averaged 1840X, 1302X, and 1415X, respectively. The sequencing depth of the variants of interest averaged 2391X (Q1–Q3: 1903–2792X).

**Figure 2:**
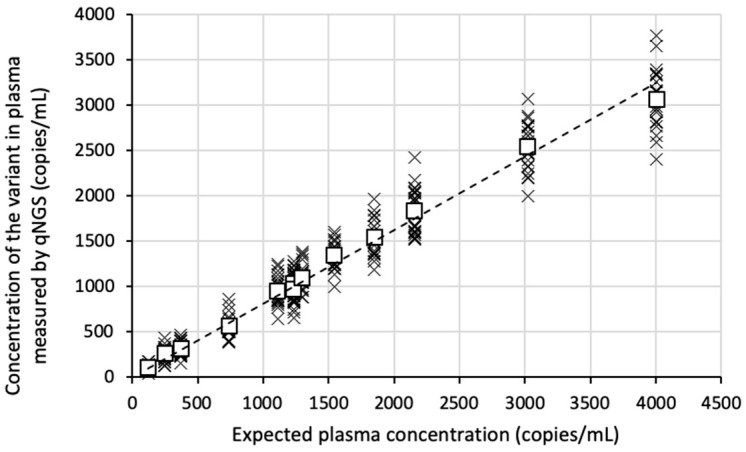
Linearity of qNGS quantification of variants in synthetic samples relative to their expected values. Normal plasma was spiked with a standard containing 13 variants detected by our NGS panel. Eight samples were prepared, and each DNA extract was analyzed three times using the qNGS method. Data points are represented by crosses, with the average concentration of each variant shown as a white square. The linear regression is shown.

Overall, qNGS accurately quantified the concentrations of all 13 variants with excellent linearity (r^2^ = 0.98; Figure 2), although there was a slight relative bias compared to the expected values (slope = 0.81).

**Figure 3:**
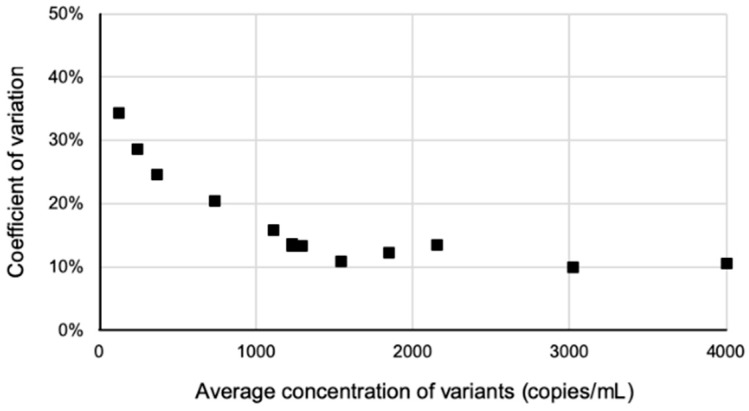
qNGS reproducibility is dependent on ctDNA concentration. Normal plasma was spiked with a standard containing 13 variants detected by our NGS panel. Eight samples were prepared, and each DNA extract was analyzed three times using the qNGS method. Coefficients of variation for each variant were measured and compared to its concentration.

The assay’s repeatability (Table 2) and reproducibility (Figure 3) improved as the concentration of the variants increased in the plasma, with observed coefficients of variation (CV) ranging from 34.3% for the least abundant variant (EGFR c.2369C > T; expected plasma concentration = 123 copies/mL) to 10.6% for the most abundant variant (CTNNB1 c.98C > A; expected plasma concentration = 4006 copies/mL).

### 3.2. qNGS Evaluation on Patient Samples

**Figure 4:**
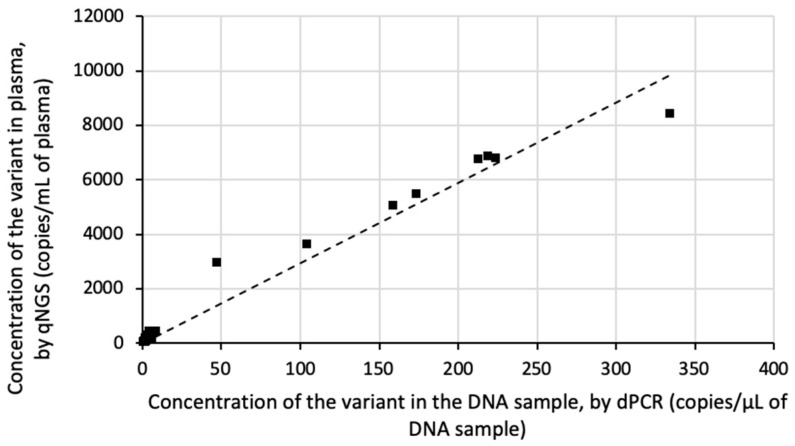
Linearity of qNGS variant quantification in pooled NSCLC patient samples relative to dPCR quantification. Eight samples were prepared by mixing, in various proportions, three plasma samples from the cancer patients positive for EGFR exon 19 deletion (c.2235_2249del), EGFR p.L858R (c.2573T > G), and BRAF p.V600E (c.1799T > A) mutations. Somatic variants were quantified by qNGS and dPCR. The linear regression is shown.

Next, we evaluated ctDNA quantification using qNGS on plasma samples from cancer patients. Positive samples were prepared by mixing three plasma samples from cancer patients, each positive for distinct mutations: EGFR exon 19 deletion (c.2235_2249del), EGFR p.L858R (c.2573T > G), and BRAF p.V600E (c.1799T > A). Eight pooled samples were created, and the somatic variants were quantified by both qNGS and dPCR. The quantification results demonstrated a highly linear correlation between the two methods (r^2^ = 0.981; Figure 4).

**Figure 5:**
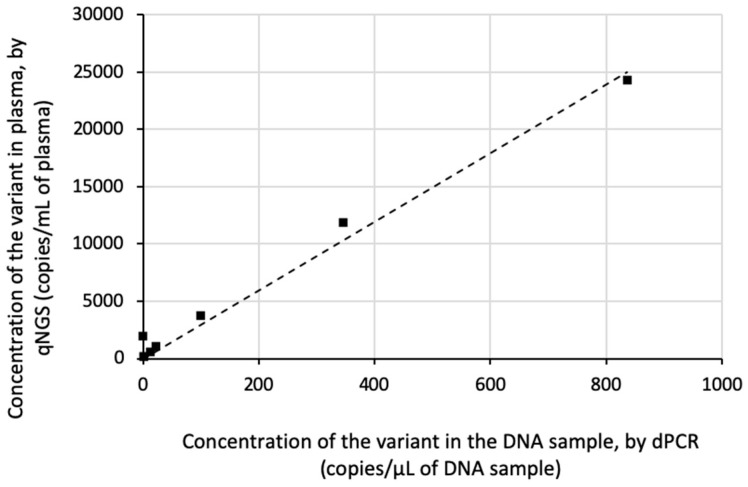
Linearity of qNGS quantification of EGFR variants in NSCLC patient samples relative to dPCR quantification. Eight plasma samples from NSCLC patients with a known EGFR exon 19 deletion in their tumors were tested using qNGS and dPCR. The linear regression is shown.

Additionally, we analyzed eight plasma samples from NSCLC patients with a known EGFR exon 19 deletion in their tumors. This alteration had been previously identified in the corresponding cell-free DNA. Again, the quantification showed a highly linear relationship between qNGS and dPCR (r^2^ = 0.991; Figure 5).

### 3.3. Clinical Application

To illustrate the use of qNGS in monitoring treatment response, we analyzed the plasma samples collected at baseline and after three weeks of treatment from four patients enrolled in the ELUCID (Early Assessment of Response to Treatment of Metastatic Lung Tumors Based on Circulating Tumor DNA) clinical trial (ClinicalTrials.gov NCT03926260). This non-controlled, prospective pilot study aims to assess the prognostic value of ctDNA kinetics for monitoring response to treatment in metastatic NSCLC. The primary objective is to determine whether changes in ctDNA concentration between baseline and week 3 can predict radiological responses to first-line treatment in advanced or metastatic NSCLC patients, regardless of the specific treatment used.

**Figure 6:**
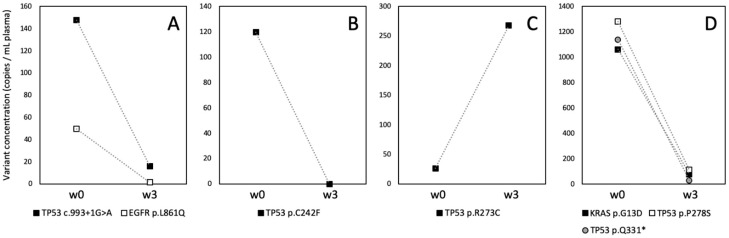
Early kinetics of ctDNA in NSCLC patients. Plasma samples collected at baseline (w0) and after three weeks of treatment (w3) from four patients ((**A**–**D**), see details in the text) enrolled in the ELUCID trial were tested using qNGS. The concentrations of the detected variants are presented.

Patient 02-035 is a 77-year-old man with a tumor in the left upper lobe and metastases to the brain and lymph nodes. His tumor harbored *EGFR* p.L861Q (p.Leu861Gln; c.2582T > A) and *TP53* (c.993 + 1G > A) mutations. Both mutations were detectable in his baseline plasma sample (w0; Figure 6A). After being treated with osimertinib, the concentrations of both mutations decreased significantly after three weeks. The first radiographic evaluation showed a partial response.

Patient 02-021 is a 71-year-old man with a tumor in the right lower lobe and lymph node metastases, carrying a rearranged *ALK* gene and *TP53* p.C242F (p.Cys242Phe; c.725G > T) mutation in his tumor. While the *TP53* mutation was detected in his plasma sample at baseline, the ALK fusion could not be detected by our NGS panel. After receiving alectinib as first-line treatment, the *TP53* mutation was no longer detectable in his plasma at week 3 (Figure 6B). The first CT evaluation showed a partial response.

Patient 02-031 is a 60-year-old woman with a tumor in the left lower lobe, and skin and brain metastases. Her tumor carried a *TP53* p.R273C (p.Arg273Cys; c.817C > T) mutation, which was detected at low concentration before treatment (Figure 6C). Following three weeks of immunotherapy with pembrolizumab, the concentration of the *TP53* mutation in plasma increased approximately 10-fold compared to baseline. Unfortunately, the patient deteriorated rapidly and passed away before the first CT scan.

Patient 01-041 is a 62-year-old man with a tumor in the lower right lobe, and metastases to the lymph nodes and adrenal glands. His tumor harbored *KRAS* p.G13D (p.Gly13Asp; c.38G > A) and two *TP53* (p.Pro278Ser; c.832C > T and p.Gln331*; c.991C > T) mutations, all of which were detected in his baseline plasma (Figure 6D). After receiving a combination of chemotherapy (carboplatin + pemetrexed) and immunotherapy (pembrolizumab), the patient showed a partial response at the first radiographic evaluation. The plasma analysis demonstrated a significant reduction in the concentration of all three mutations at week 3.

## 4. Discussion

Most studies evaluating the value of ctDNA for monitoring treatment effectiveness focus on variations in VAF. However, different approaches have been used to assess these variations, often with thresholds set arbitrarily. For instance, some studies require a decrease in VAF between baseline and treatment evaluation [16], with a 50% reduction in VAF frequently used as a benchmark for patients undergoing targeted therapy [17], chemotherapy, or immunotherapy [18,19]. In certain cases, the complete clearance of ctDNA is considered an endpoint [20]. However, comparing VAFs across studies is challenging since VAFs do not account for variations in cfDNA concentration unrelated to tumor pathology.

To address these limitations, we developed a quantitative NGS method for detecting ctDNA variants. This approach, combining UMIs and QSs, produces results that align linearly with dPCR, the established gold standard for absolute quantification of variants. When analyzing plasma samples spiked with DNA, qNGS showed a slight underestimation of variant concentrations by an average of 19% compared to expected values. This modest bias could be attributed to minor reconstitution issues or the potential degradation of the OncoSpan reference standard before the lysis solution was added.

Few protocols attempting to estimate variant concentration by NGS have been published. Hoerres et al. described a method that uses synthetic oligonucleotides [21,22] to normalize quantification of the Epstein–Barr virus (EBV) genome in plasma [23]. They demonstrated good linearity when compared to dPCR. In our protocol, the use of UMIs allowed for the counting of sequenced molecules without the interference of PCR duplicates, while the inclusion of QSs normalizes qNGS quantification to a highly comparable reference. QSs must be tailored to both the library preparation technology and the panel used. In this study, we employed three QSs that were analogous to three reference loci targeted by our panel, and specifically designed to be amplified by our library preparation method, which is based on the SPE technology. This method uses a single primer to select target regions. If AMP primers in the panel differ, the QSs’ sequence will need to be adjusted. Additionally, the general construction of QSs must be modified for use with other library preparation methods, such as conventional amplicon-based or capture-based approaches. Using a greater number of QSs could improve the accuracy of genomic equivalent estimation in samples, leading to more precise variant quantification.

The presence of somatic copy number variations of a gene of interest can interfere with absolute quantification by qNGS. The qNGS calculation method is based on the assumption that each genome in the circulating DNA contains only two copies of the gene of interest. If there is a somatic amplification of a gene, this assumption is violated, which may lead to an underestimation of the variant measurement. From a clinical perspective, gene amplification is problematic regardless of the assay method, because it alters the correlation between ctDNA quantification and tumor volume, which may lead to inappropriate clinical decisions. Therefore, it is crucial to document copy number alterations in tumor tissue whenever possible, to avoid using an amplified variant allele to quantify ctDNA. Additionally, it is recommended to use variants from independent genes for ctDNA quantification to provide a more accurate assessment of ctDNA levels.

Our protocol was evaluated under specific experimental conditions. The standard sequencing and bioinformatics analysis parameters we used allow us to detect mutations present at an allele frequency of 0.5–1% or higher. Among the samples from the Oncospan standard, the detected allele frequencies ranged from 0.8% (EGFR p.T790M mutation) to 22.4% (CTNNB1 p.S33Y mutation). We therefore consider this to be the valid range for our approach. Improving the sensitivity of an NGS approach requires increasing the amount of DNA analyzed, as well as sequencing depth and optimized bioinformatics analysis parameters [24,25,26]. Further work will be needed to evaluate our approach for low variant loads.

Quantitative NGS offers significant potential for improving ctDNA monitoring in the non-invasive surveillance of tumor volume and treatment response. It enables the monitoring of ctDNA variations independently of changes in non-tumor circulating DNA, thus reducing the risk of misinterpreting ctDNA kinetics [8,9,10]. Additionally, qNGS has a key advantage over targeted analyses, such as dPCR, because it does not require prior knowledge of the specific genetic alterations present in the tumor to explore ctDNA. This makes the technique more broadly applicable without the need for patient-specific customization, provided that the gene panel is sufficiently comprehensive. Moreover, qNGS enables the simultaneous quantification of multiple variants, which is particularly beneficial. Monitoring only a single variant carries the risk of tracking a tumor sub-clone that may not be representative of the entire tumor [27]. By incorporating several somatic variants into ctDNA monitoring, qNGS provides a more accurate reflection of tumor burden variations and facilitates the detection of emerging treatment-resistant variants. The examples of patients from the ELUCID trial demonstrate the advantages of this quantitative approach. In these cases, tumor genotypes had been previously determined, which was helpful in the development of the test. However, even without prior genotype information, the results could have been interpreted in a similar manner.

## 5. Conclusions

We have developed an innovative method for the absolute quantification of ctDNA via NGS, overcoming the limitations of variations in non-tumor circulating DNA. Our proof of concept, demonstrated using samples from a small cohort of patients, shows the method’s potential for broad clinical application. This technique is now being employed to analyze plasma samples from the full ELUCID trial cohort (100 patients), providing a unique opportunity to evaluate whether early ctDNA kinetics can serve as a reliable predictor of treatment response, irrespective of the treatment modality. By eliminating the reliance on individual tumor genotypes, our method offers a more standardized and comprehensive approach to ctDNA monitoring, which could significantly enhance non-invasive tumor surveillance and response assessment in clinical practice.

## Figures and Tables

**Table 1:** Sequencing primers targeting both the QSs and their reference loci.

NGS Primer	Sequence
QS1	GCCTAAATGCTCCACTTAAAAGCTAAAGATGACA
QS2	AAAAATGGGCGGAGGAGAGTAGTCTGAATT
QS3	CCAGTGTTGTGGGATATTAATGTGCATTACATAG

**Table 2:** List of variants of the OncoSpan reference standard. Concentration measured by qNGS in the reconstituted samples and repeatability of the test.

Variant	Concentration (Copies/mL)	Coefficient of Variation
NRAS p.Q61K (p.Gln61Lys; c.181C > A)	1540.8	10.1%
CTNNB1 p.S33Y (p.Ser33Tyr; c.98C > A)	4006.1	10.4%
CTNNB1 p.S45del (p.Ser45del; c.133_135del)	1232.7	12.0%
PIK3CA p.E545K (p.Glu545Lys; c.1633G > A)	1109.4	14.5%
PIK3CA p.H1047R (p.His1047Arg; c.3140A > G)	2157.2	11.2%
KIT p.D816V (p.Asp816Val; c.2447A > T)	1232.7	13.4%
EGFR p.G719S (p.Gly719Ser; c.2155G > A)	3020.0	8.9%
EGFR p.E746_A750del (p.Glu746_Ala750del; c.2235_2249del)	246.5	25.1%
EGFR p.T790M (p.Thr790Met; c.2369C > T)	123.3	31.9%
EGFR p.L858R (p.Leu858Arg; c.2573T > G)	369.4	23.8%
BRAF p.V600E (p.Val600Glu; c.1799T > A)	1295.7	10.2%
KRAS p.G13D (p.Gly13Asp; c.38G > A)	1849.0	11.0%
KRAS p.G12D (p.Gly12Asp; c.35G > A)	739.6	17.2%

## Data Availability

The data presented in this study are available on request from the corresponding author.

## Supplementary Materials

The following supporting information can be downloaded at https://www.mdpi.com/article/10.3390/cancers17050783/s1, Figure S1: Detailed sequences of the QSs, along with their reference loci; Table S1: Target regions of the QIAseq targeted DNA custom panel.
