# Supplementary material for: Absolute Quantification of Nucleotide Variants in Cell-Free DNA via Quantitative NGS: Clinical Application in Non-Small Cell Lung Cancer Patients
Source: Cancers (Basel). 2025 Feb 25;17(5):783. doi: 10.3390/cancers17050783 (PMC11898635; doi:10.3390/cancers17050783)
Supplement: Supplementary file 1 [file cancers-17-00783-s001.zip › cancers-3403392-supplementary.pdf]

Quantification standard 1 (QS1)
Reference locus: Human GRCh38 (hg38) chr12: 10,085,326 – 10,085,428
Reference locus sequence<br>gcctaaatgctccacttaaaagctaaagatgaca-------------------------gaatggatttcaccaatcaagtatctgctgtcttcaaaagactcacctaacacataaggactcacataa
QS sequence<br>GTGACATCTACGGTGATCCGACATCTCCTGgcctaaatgctccacttaaaagctaaagatgacaGATTACAACACGAGTTCGACCGCGTgaatggatttcaccaatcaagtatctgctgtcttcaaaagactcacctaacacataaggactcacataaGTTGTTAGCATCGCCGTCATATCGCAAGGCAT
**Quantification standard 2 (QS2)**
Reference locus: Human GRCh38 (hg38) chr14: 20,343,068 – 20,343,170
Reference locus sequence<br>aaaaatgggcggaggagagtagtctgaatt-------------------------gggttatgaggtcccctgcggggtacctcacctcagccattgaactcacttcgctggccgtgagtctgttcca
QS sequence<br>GTGACATCTACGGTGATCCGACATCTCCTGaaaaatgggcggaggagagtagtctgaattGATTACAACACGAGTTCGACCGCGTgggttatgaggtcccctgcggggtacctcacctcagccattgaactcacttcgctggccgtgagtctgttccaGTTGTTAGCATCGCCGTCATATCGCAAGGCAT
**Quantification standard 3 (QS3)**
Reference locus: Human GRCh38 (hg38) chr17: 75,738,768 – 75,738,870
Reference locus sequence<br>ccagtgttgtgggatattaatgtgcattacatag-------------------------gcaaggagttatgtgatcaaataggtttgagctaggcacggctgcgcgtgcctgtagtcccagctactc
QS sequence<br>GTGACATCTACGGTGATCCGACATCTCCTGccagtgttgtgggatattaatgtgcattacatagGATTACAACACGAGTTCGACCGCGTgcaaggagttatgtgatcaaataggtttgagctaggcacggctgcgcgtgcctgtagtcccagctactcGTTGTTAGCATCGCCGTCATATCGCAAGGCAT

Suppl. Figure S1. Detailed sequences of the QSs, along with their reference loci. Green: sequence targeted by the NGS panel primers; Red: QS-specific insertion; Blue: generic ends; Orange: sequence targeted by the dPCR reverse primer for specific quantification of each QS.

Suppl. Table S1. Target regions of the QIAseq Targeted DNA Custom Panel.

Gene	Target regions	Transcript
AKT1	exon 3	NM_001014431.2
ALK	exons 22 to 25	NM_004304.5
BRAF	exons 11, 15	NM_004333.6
CTNNB1	exon 3	NM_001904.4
EGFR	exons 18 to 21	NM_005228.5
ERBB2	exons 19 to 22	NM_004448.4
ERBB4	codons 393, 452	NM_005235.3
FGFR2	codons 252, 549, 659	NM_000141.5
FGFR3	exons 6, 8, 13	NM_000142.5
HRAS	exons 2, 3, 4	NM_005343.4
IDH1	codons 100, 132	NM_005896.4
IDH2	codon 172	NM_002168.4
KIT	exons 8, 9, 11, 13, 14, 17, 18	NM_000222.3
KRAS	exons 2, 3, 4	NM_033360.4
MAP2K1	exon 2	NM_002755.4
MET	intron 13, exon 14	NM_001127500.3
NRAS	exons 2, 3, 4	NM_002524.5
PDGFRA	exons 12, 14, 18	NM_006206.6
PIK3CA	exons 10, 21	NM_006218.4
RET	exons 11, 16	NM_020975.6
TP53	exons 2 to 11	NM_000546.6
